# Exploring conversation coordination in patients with schizophrenia

**DOI:** 10.1192/j.eurpsy.2025.2210

**Published:** 2025-08-26

**Authors:** M. Champagne-Lavau, C. Petrone, A. Poulet, C. Faget, C. Lançon

**Affiliations:** 1 CNRS, LPL, Aix-Marseille University, Aix-en-Provence; 2 Department of speech language pathology, Claude Bernard Lyon1 University, Lyon; 3 Department of Psychiatry, AP-HM: La Conception University Hospital, Marseille, France

## Abstract

**Introduction:**

Individuals with schizophrenia (SZ) are known to be impaired in their social and communication abilities. However, these impairments are not well characterized. More specifically, little is known about how SZ individuals take into account their interlocutor during conversation. Verbal backchannels (e.g., okay, yes) have been described as crucial cues that contribute to conversation coordination by allowing the updating of knowledge shared between interlocutors (Gravano & Hirschberg, 2011, Comput. speech lang.*, 25*, 601-634). They could reflect the ability of interlocutors to take into account their partner’s perspective during conversation.

**Objectives:**

The aim of the present study was to explore how SZ individuals manage conversation coordination with their interlocutor.

**Methods:**

Thirty-one SZ participants and 30 healthy control (HC) participants matched for age and educational level performed a referential communication task with a partner (i.e., a collaborative game; Champagne-Lavau et al., 2009, Cogn. Neuropsychiatry, 14, 217-239.). During this game, they played either the role of Director (condition 1) or the role of Addressee (condition 2) with an experimenter. In condition 1, we performed prosodic analyses on the cues known to predict the production of a backchannel (i.e., backchannel-inviting cues, Gravano & Hirschberg, 2011) by the Addressee (e.g., duration and intonational contour of the Director’s utterance produced before the backchannel). In condition 2, we performed phonetic analyses (e.g., f0min, f0max, pitch span, duration) on the backchannels (i.e., yes) produced by the Addressee. SZ participants’ severity of symptoms was measured using the PANSS. Participants were also assessed on their theory of mind abilities with the Hinting task.

**Results:**

Data from 22 SZ and 17 HC participants were analyzed. The main results did not show any difference between SZ and HC participants regarding the production of backchannel-inviting cues (condition 1) and regarding the number of backchannels produced (condition 2). However, phonetic analyses in condition 2 showed that SZ participants produced backchannels with a shorter duration (222 ms ± 85) and a reduced pitch span (0.443 ± 0.301) compared to HC participants (duration: 265 ms ± 91; pitch span: 0.586 ± 0.367). We also found a correlation between pitch span and PANSS (general score) (r = -0.467, p = 0.029) and a correlation marginally significant between pitch span and theory of mind abilities (r = 0.395, p = 0.069).

**Conclusions:**

This exploratory study seems to show that SZ participants’ production of backchannels (reflecting their role in conversation coordination) is related to their theory of mind abilities and to their symptoms.

**Disclosure of Interest:**

None Declared

